# MIP-T3 Expression Associated with Defects of Ciliogenesis in Airway of COPD Patients

**DOI:** 10.1155/2020/1350872

**Published:** 2020-02-10

**Authors:** Wen-Jun Wang, Shi-Fang Yang, Zhi-Rui Gao, Ze-Ru Luo, Yuan-Ling Liu, Xing-Lin Gao

**Affiliations:** ^1^Department of Respiratory and Critical Care Medicine, Guangdong Provincial People's Hospital (Guangdong Academy of Medical Sciences), Guangdong Provincial Geriatrics Institute, First Clinical Medicine Institute of South China University of Technology, Guangzhou, Guangdong, China; ^2^Department of Respiratory and Critical Care Medicine, Yan'an Hospital of Kunming, Kunming, China

## Abstract

*Introduction*. Some studies have found that cilia were shorter in COPD smokers than in nonsmokers or healthy smokers. However, the structural abnormalities of cilia and the cause of such abnormalities in COPD patients still remain unknown. Tumor necrosis factor alpha receptor 3 interacting protein 1 (MIP-T3) may play an important role in the progress of ciliary protein transporting.

**Objectives:**

This study aimed at exploring the dominated structural abnormalities of cilia and the involvement of MIP-T3 in the pathogenesis of cilia of COPD patients.

**Methods:**

Patients who accepted pulmonary lobectomy were divided into 3 groups: the chronic obstructive pulmonary disease (COPD) smoker group, the healthy smoker group, and the nonsmoker group, according to smoking history and pulmonary function. The ultrastructure of cilia and the percentage of abnormal cilia were analyzed using a transmission electron microscope. Real-time PCR, immunohistochemical staining, and western blotting in bronchial epithelium were used to determine MIP-T3 mRNA and protein expression. The relationship between the percentage of abnormal cilia and lung function and MIP-T3 protein expression was analyzed.

**Results:**

Patients in the COPD smoker group had increased percentage of abnormal cilia comparing to both the healthy smoker group and the nonsmoker group (both *P* values <0.05). MIP-T3 expression was significantly declined in the COPD smoker group (*P* values <0.05). MIP-T3 expression was significantly declined in the COPD smoker group (*P* values <0.05). MIP-T3 expression was significantly declined in the COPD smoker group (*P* values <0.05). MIP-T3 expression was significantly declined in the COPD smoker group (

**Conclusions:**

Our results suggested that the abnormal ciliary ultrastructure, which was common in COPD patients, might be due to MIP-T3 downregulation.

## 1. Introduction

Chronic obstructive pulmonary disease (COPD) remains a significant public health problem causing a considerable socioeconomic burden [1]. Although the corresponding pathogenesis has not been determined to date, mucus cilia clearance function damage played an important role [2]. Cilia are evolutionarily conserved microtubule-based organelles protruding from the cell surface and widely exist in prokaryotes and eukaryotes. Recent studies have shown that cilia served important functions in motility, sensory reception, signaling, and immune function [3]. Hogg confirmed that cilia were shorter in COPD smokers than in nonsmokers or healthy smokers, and they also found significant correlations between cilia length and FEV_1_, FEV_1_/FVC among smokers (both healthy and with COPD) [4]. However, previous studies mainly focused on cilia length rather than the ultrastructure. The dominated structural abnormalities of cilia in COPD patients and the cause of such structural abnormalities still remain unknown. Tumor necrosis factor alpha receptor 3 interacting protein 1 (MIP-T3) may play an important role in the progress of ciliary protein transporting [5, 6]. A study found that MIP-T3 mRNA was expressed in all human tissues, such as bronchus, lungs, testis, ovary, spinal cord, thyroid gland, and prostate [7]. However, whether MIP-T3 plays a critical role in cilium biogenesis of COPD patients is unclear. In the present study, we aimed to explore the dominated structural abnormalities of cilia and the involvement of MIP-T3 in the pathogenesis of cilia of COPD patients.

## 2. Materials and Methods

### 2.1. Subjects

Patients were divided into three groups according to smoking history and lung function: the chronic obstructive pulmonary disease (COPD) smoker group, the healthy smoker group, and the nonsmoker group. COPD patients, diagnosed according to the 2016-updated Global Initiative for Chronic Obstructive Lung Disease (GOLD), who had a smoking index of more than 10 pack years, were categorized into the COPD smoker group. The healthy smoker group included those who smoked more than 10 pack years, but with normal lung functions, and the nonsmoker group with no smoking history. People with the following chronic conditions were excluded from the study. Such chronic conditions included bronchiectasis, pneumonia, bronchial asthma, autosomal dominant polycystic kidney disease, cystic fibrosis, Kartagener syndrome, internal transfer of primary cilia dyskinesia disease, retinal degeneration, olfactory dysfunction, and infertility. Patients currently undergoing radiotherapy, chemotherapy, adrenal cortex hormone, theophylline, anticholinergic drugs, beta blockers, and catecholamine, which could affect bronchial cilia function in 2 months, were excluded. Additional exclusion criteria were some specific constitutions or with severe cardiopulmonary liver and kidney disease.

Bronchial epithelium samples were collected from 56 patients who accepted pulmonary surgery in Guangdong Provincial People's Hospital between February and November in 2016. Details of subjects' characteristics are described in Table 1. The bronchial epithelium samples were collected and analyzed by three different methods, including real-time PCR and western blotting, where the samples were frozen immediately in liquid nitrogen, electron microscopy, where the samples were fixed in 2.5% glutaraldehyde, and immunohistochemistry staining, where the samples were fixed in 10% paraformaldehyde. The study was performed in accordance with the Declaration of Helsinki, International Conference on Harmonisation: Harmonised Tripartite Guideline for Good Clinical Practice, and local regulations and was approved by the Ethics Committee of Guangdong Provincial People's Hospital Guangdong Academy of Medical Sciences, Guangzhou, China. Written informed consent was obtained from patients.

### 2.2. Electron Microscopy

Bronchial epithelium samples were fixed in 2.5% glutaraldehyde as previously described. The samples were sent to College of Pharmacy, electron microscope room, Southern Medical University, Guangzhou, China, for transmission electron microscope (TEM) analysis. Simply, samples were postfixed using 1% osmium tetroxide for 2 hours and then were scraped, pelleted, dehydrated, infiltrated, and embedded. Subsequently, ultrathin sections were cut and stained with uranyl acetate. Under the multiples of 40000x, the total number of cilia and the number of abnormal cilia were counted, and the percentage of abnormal cilia was calculated. Cilia defects included cilia membrane blisters, microtubules defects, compound cilia, and giant cilia [8, 9].

### 2.3. Quantitative Real-Time Polymerase Chain Reaction

Total RNA was extracted from bronchial epithelium using Trizol reagent (Invitrogen, Shanghai, China), following the manufacturer's protocols. 1 *μ*g of the extracted RNA was transcribed into cDNA using a cDNA synthesis kit (Thermo, USA). The relative expression of MIP-T3 to GAPDH was measured by the real-time PCR using a SYBR green-based RT-PCR kit (Thermo, USA) and specific primers (Takara, Dalian, China). The forward and reverse primer sequences were sense 5′-CCTGCTGGCCAAGATAAGTCTGA-3′ and antisense 5′-TGTAGCGCCTCCATGCTGTC-3′ for MIP-T3 and sense 5′-GCACCGTCAAGGCTGAGAAC-3′ and antisense 5′-GGTGAAGACGCCAGTGGA-3′ for GAPDH. The cycling conditions were denatured for 20 minutes at 94°C. 40 cycles of amplification were performed at 94°C for 30 seconds, 58°C for 30 seconds, and 72°C for 30 seconds. The relative gene expression of MIP-T3 was calculated by 2^−△△CT^.

### 2.4. Immunohistochemical Staining

Bronchial epithelium samples were embedded into paraffin, and 5 *μ*m horizontal sections were cut in a paraffin slicing machine. Samples were dewaxed, washed with water, and then repaired antigen and treated in 3% H_2_O_2_ for 25 minutes to deactivate endogenous peroxidase. The samples were blocked with 5% fat-free milk for 1 hour and then incubated with primary antibodies (anti-MIP-T3 rabbit polyantibody, diluted 1:200; OriGene, MA, USA) overnight at 4°C in a humidified chamber. They were subsequently incubated with a solution of the goat anti-rabbit IgG HRP-conjugated antibody (diluted 1:200; CWBIO, Guangzhou, China) for 1 hour at room temperature. After chromogenic reaction with DAB staining, the samples were dehydrated, cleared in xylene, and covered with neutral balsam. Image-Pro plus 6.0 software was used to measure mean IOD of the MIP-T3 expression in bronchial epithelium.

### 2.5. Western Blotting

Total protein of bronchial epithelium samples was extracted by RIPA buffer (Beyotime, Nantong, China) according to the manufacturer's protocols. After determining concentration by the BCA protein assay kit (Beyotime, Nantong, China), the protein was denatured and stored at −20°C. Samples containing 60 *μ*g protein were subjected to 8% SDS-PAGE electrophoresis and transferred to a PVDF membrane (Weijia, Guangzhou, China). The membrane was blocked with 5% fat-free milk for 1 hour and then blotted with primary antibodies (anti-MIP-T3 rabbit polyantibody, diluted 1:500; OriGene, MA, USA, and anti-GAPDH rabbit polyantibody, diluted 1:2000; CWBIO, Guangzhou, China) overnight at 4°C. They were subsequently incubated with a solution of the goat anti-rabbit IgG HRP-conjugated antibody (diluted 1:5000; CWBIO, Guangzhou, China) for 1 hour at room temperature. Finally, the membranes were processed using the ECL chemiluminescence reaction (Beyotime, Nantong, China) and followed on the RM2016 imaging system. The band density was measured by Image J, and the relative expression of the target protein was compared with GAPDH.

### 2.6. Statistical Analysis

Results were expressed as the mean ± SD. Statistical significance of the differences between experimental groups was calculated using one-way ANOVA with Bonferroni post-test. Differences in proportions between groups were examined by a chi-square test. The Spearman or Pearson test was used to determine correlations. Results with a *P* value less than 0.05 were considered as significant. Statistical analysis was performed by SPSS version 20.0 for Windows (IBM Corp., Armonk, NY), and figures were produced in GraphPad prism 5 (GraphPad, San Diego, USA).

## 3. Results

### 3.1. Patient Characteristics

As shown in Table 1, there was no significant difference in sex, age, and composition of diseases among groups. Comparing to the control group, the smoking index in the COPD group and the smoking group was significantly higher (*P* value <0.05). Also, there was significant difference between the COPD smoker group and the healthy smoker group (*P* value <0.05). In contrast with the nonsmoker group and the healthy smoker group, lung functions in the COPD smoker group were significantly declined (*P* value <0.05). Moreover, there was no significant difference between the control group and the smoking group.

### 3.2. Comparison of Abnormal Cilia between the Three Groups

**Figure 1:**
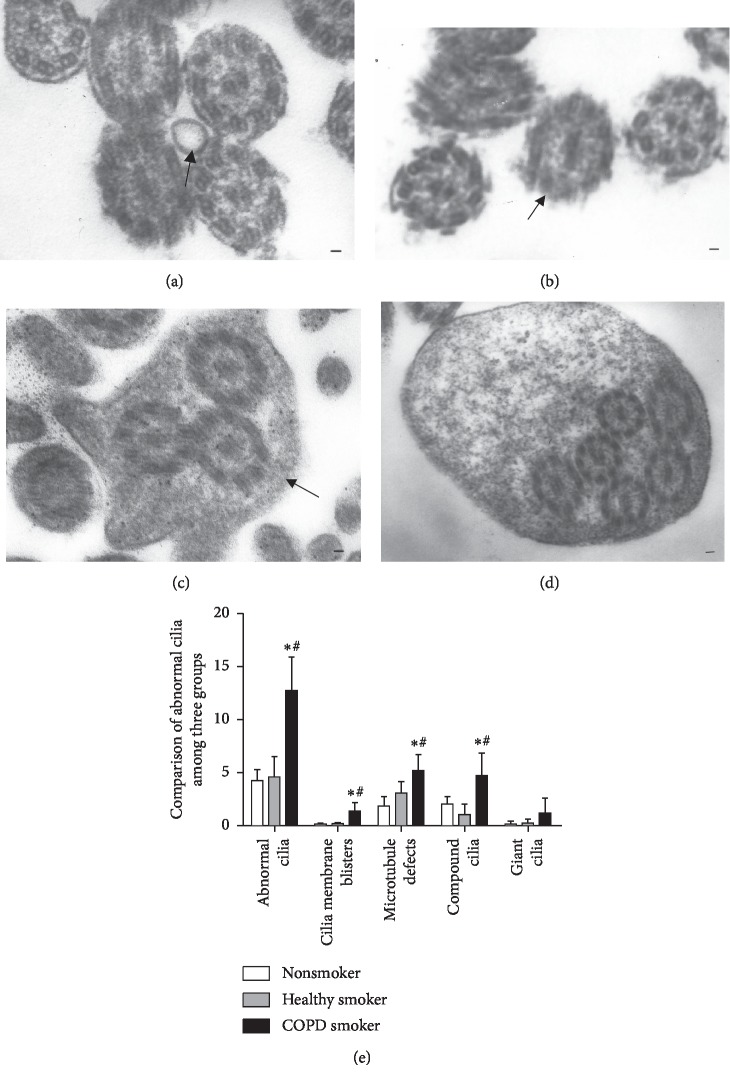
The abnormal cilia transmission electron microscope. Cilia membrane blisters (a), microtubules defects (b), compound cilia (c), and giant cilia (d), black lines represented 5 *μ*m. Comparison of the percentage of abnormal cilia among three groups (e). Data were expressed as mean ± SD, ^*∗*^*P* value <0.05 vs nonsmoker group, ^#^*P* value <0.05 vs healthy smoker group.

Cilia membrane blisters (Figure 1(a)), microtubules defects (Figure 1(b)), compound cilia (Figure 1(c)), and giant cilia (Figure 1(d)) were observed by TEM. As shown in Figure 1(e), the percentage of abnormal cilia in the COPD smoker group (12.74 ± 3.17) was significantly higher than the healthy smoker group (4.61 ± 1.90) and the nonsmoker group (4.24 ± 1.07) (*P* value <0.05). No difference was observed between the healthy smoker group and the nonsmoker group (*P* value >0.05). In addition, the percentage of cilia membrane blisters, microtubules defects, and compound cilia was higher in the COPD smoker group (1.38 ± 0.82, 5.20 ± 1.52, 4.75 ± 2.12) than the healthy smoker group (0.19 ± 0.12, 3.08 ± 1.09, 1.04 ± 1.01 ) and the nonsmoker group(0.15 ± 0.13, 1.86 ± 0.89, 2.05 ± 0.69 ) (*P* values <0.05). Giant cilia did not differ significantly among three groups (*P* value >0.05). In conclusion, these results verified that the percentage of cilia defects increased in patients with COPD.

### 3.3. Correlation Analysis between Cilia Defects and Lung Function Index

The correlationship between the percentage of abnormal cilia and lung function was performed by Pearson correlation analysis. The results found that the percentage of abnormal cilia of COPD was inversely correlated with FEV_1_, FEV_1_/FVC, and FEV_1_%pred (*r* = −0.756, −0.684, −0.789, respectively; all *P* values < 0.05), not with FVC (*P* value > 0.05).

### 3.4. Expression of MIP-T3 in Bronchial Epithelium

**Figure 2:**
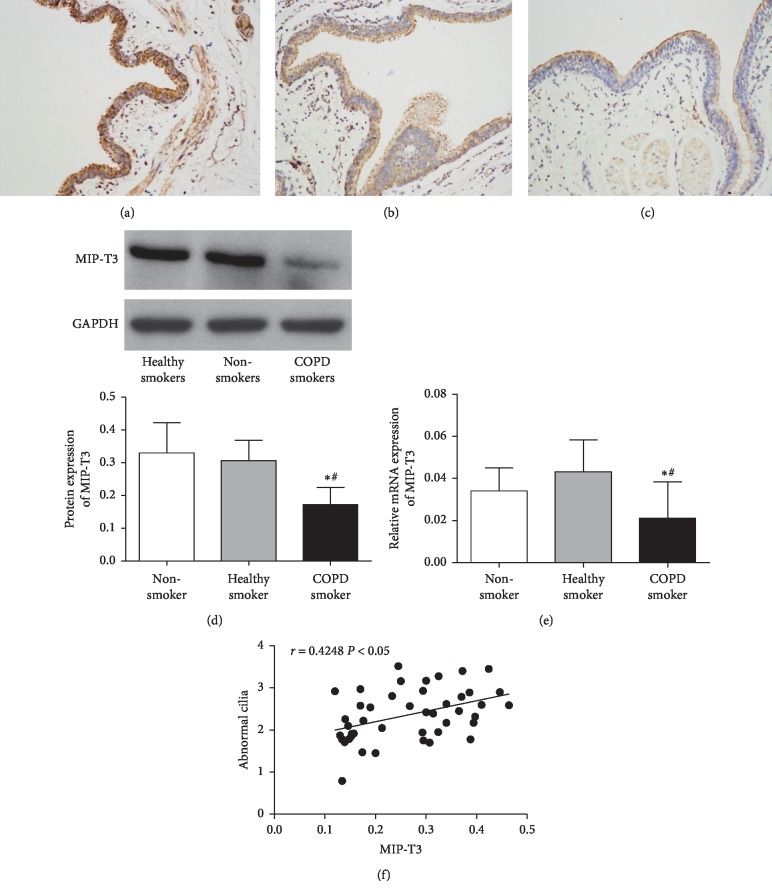
MIP-T3 expression in bronchial epithelium. Immunohistochemistry analysis of the MIP-T3 protein expression in epithelial cells and cilia of nonsmokers (a), healthy smokers (b), and COPD smokers (c), black lines represented 200 *μ*m. The level of MIP-T3 protein and mRNA expression was determined by western blotting and real-time PCR (d,e). The correlation analysis between the MIP-T3 protein expression and the percentage of abnormal cilia was shown (f). Data were expressed as mean ± SD, ^*∗*^*P* value < 0.05 vs nonsmoker group, and ^#^*P* value < 0.05 vs healthy smoker group.

As shown in Figures 2(a)–2(c), MIP-T3 was expressed on bronchial epithelium cells and cilia. MIP-T3 mRNA and protein expression in COPD smokers (0.017 ± 0.007, 0.165 ± 0.035) declined significantly compared with nonsmokers (0.038 ± 0.003, 0.309 ± 0.041) and healthy smokers (0.043 ± 0.005, 0.325 ± 0.013) (Figures 2(d) and 2(e), *P* values <0.05). No difference was observed between the healthy smoker group and the nonsmoker group (*P* value >0.05). Further correlation analysis revealed that the MIP-T3 protein expression was positively correlated with the percentage of abnormal cilia (*r* = 0.4248, *P* value <0.05, Figure 2(f)).

## 4. Discussion

Chronic obstructive pulmonary disease (COPD) is characterized by persistent respiratory symptoms and airflow limitation and remains a significant public health problem with a considerable socioeconomic burden [1]. Although the corresponding pathogenesis has not been determined to date, both mucus cilia clearance function damage and mucociliary clearance apparatus played an important role [2]. Previous studies mainly focused on cilia length. A lot of experiments suggested that defects of cilia length were related to both smoking and defects of mucociliary clearance in COPD patients [3, 10]. However, there are few studies on the normal structure of cilia.

Our experiment indicated that compared with the smoking group and the control group, the percentage of cilia ultrastructure abnormalities in the COPD group was significantly higher. The major structural abnormalities of cilia in COPD were microtubule defects, compound cilia, and cilia membrane blister. Moreover, our experiment indicated that the abnormal cilia of COPD were inversely correlated with FEV_1_, FEV_1_/FVC, and FEV_1_%pred. These results suggested that defects of cilia may involve in the pathogenesis and development of COPD. Compared with previous studies, the percentage of abnormal cilia in our research were slightly lower [11, 12]. The divergence may be due to the source of the sample. Our specimens were from patients who accepted lung lobectomy and had lung function index tolerable. There was no significant difference of cilia ultrastructure abnormalities between the smoking group and the control group. Contrary to our findings, Verra et al. [13] reported that cilia ultrastructural abnormalities were higher in smokers (16.5%) and ex-smokers (17.5%) than in nonsmokers (5.2%) or control subjects (0.7%).

Microtubules are the main components of the cilia. Tumor necrosis factor receptor-associated factor 3 interacting protein 1 (MIP-T3), 83kD, is a microtubule binding protein. Previous studies have shown that MIP-T3 might play an important role in cilia formation, function maintenance, and depolymerization [14, 15]. Hessel et al. found that the MIP-T3 gene expression was declined in COPD smokers [8]. However, experiments were limited to the MIP-T3 gene expression rather than the protein. Our study indicated that both MIP-T3 gene and protein expression were downregulated in the COPD patients, which was consistent with the Hessel study. Furthermore, MIP-T3 was positively correlated with the percentage of abnormal cilia. These data suggested that MIP-T3 may be crucial for cilium biogenesis of COPD. The reason for the normal cilia structure may be MIP-T3 affects the assembly of cilia, as MIP-T3 acts as a constituent protein of the subunit of the intraflagellar transport complex, and participates in the cilia axis by transporting the cilia precursors synthesized in the cytoplasm into the cilia [16, 17].

In summary, cilia ultrastructure abnormalities, including microtubule defects, compound cilia, and cilia membrane blister, were found in COPD patients. MIP-T3 expression was downregulated in COPD, and MIP-T3 was positively correlated with the percentage of abnormal cilia. We recognized that our study had limitations, such as lack of research on the mechanism of MIP-T3 leading to the abnormal cilia structure and the role of MIP-T3 in the development of COPD. Therefore, further studies should be warranted to address these questions.

## Figures and Tables

**Table 1:** Subject characteristics.

	Nonsmoker group	Healthy smoker group	COPD smoker
Total of subjects (*n*)	21	17	18
Sex (male, *n* (%))	18 (85.71)	16 (94.11)	17 (94.44)
Age (year, mean ± SD)	58.57 ± 11.23	57.35 ± 10.40	62.22 ± 9.40
Smoking index (pack years, mean ± SD)		28.91 ± 18.49	46.37 ± 17.66^#^
Disease constitution carcinoma, *n* (%)	19 (90.47)	16 (94.11)	17 (94.44)
FEV_1_(*L*, mean ± SD)	2.30 ± 0.78	2.69 ± 0.63	1.79 ± 0.47^*∗*#^
FVC (*L*, mean ± SD)	2.97 ± 0.98	3.58 ± 0.87	3.04 ± 0.51
FVC%pred (mean ± SD)	84.24 ± 6.62	86.74 ± 7.01	81.52 ± 5.22
FEV_1_/FVC (mean ± SD)	78.29 ± 6.78	75.53 ± 8.10	58.33 ± 10.23^*∗*#^
FEV_1_%pred (mean ± SD)	96.76 ± 10.10	94.12 ± 12.92	65.89 ± 15.44^*∗*#^

FEV_1_: forced expiratory volume in the first second; FVC: forced vital capacity. ^*∗*^vs nonsmoker group, *P* value <0.05. ^#^vs healthy smoker group, *P* value <0.05.

## Data Availability

The data used to support the findings of this study are available from the corresponding author upon request.
